# Correlation between coronary artery calcification and COVID-19

**DOI:** 10.22088/cjim.15.3.466

**Published:** 2024-08-01

**Authors:** Saeed Abrotan, Seyed Farzad Jalali, Mohammadtaghi Hedayati-godarzi, Iraj Jafaripour, Mehrdad Saravi, Naghmeh Ziaie, Roghayeh Pourkia, Kamyar Amin, Ali Bijani, Masomeh Bayani, Sorayya Khafri, Milad Bakhshi, Saeed Kargar-Soleimanabad, Erfan Ghadirzadeh

**Affiliations:** 1 Department of Cardiology, School of Medicine, Ayatollah Rouhani Hospital, Babol University of Medical Sciences, Babol, Iran; 2 Social Determinants of Health Research Center, Health Research Institute, Babol University of Medical Sciences, Babol, Iran; 3 Infectious Diseases and Tropical Medicine Research Center, Health Research Institute, Ayatollah Rouhani Hospital, Babol University of Medical Sciences, Babol, Iran; 4 Infertility and Reproductive Health Research Center, Health Research Institute, Babol University of Medical Sciences, Babol, Iran; 5 Student Research Committee, Faculty of Medicine, Babol University of Medical Sciences, Babol, Iran; 6 Student Research Committee, Faculty of Medicine, Mazandaran University of Medical Sciences, Sari, Iran

**Keywords:** Coronary heart disease, COVID-19, Hospitalization, Morbidity, Mortality, Coronary artery calcification

## Abstract

**Background:**

Coronary heart disease (CHD) is an underlying cardiac condition contributing to increased COVID-19 mortality and morbidity which can be assessed by several diagnosis methods including coronary artery calcification (CAC). The goal of this study was to find out if there were potential links between CAC, clinical findings, severity of COVID-19, and in-hospital outcomes.

**Methods:**

This retrospective study evaluated 551 suspected patients admitted to teaching hospitals of the Babol University of Medical Sciences, Babol, Iran, from March to October 2021. Data included previous diseases, comorbidities, clinical examinations, routine laboratory tests, demographic characteristics, duration of hospitalization, and number of days under ventilation were recorded in a checklist.

**Results:**

Findings of current study provide evidence of a significant relationship between coronary artery calcification (CAC) and in-hospital mortality. Additionally, we observed significant correlations between CAC and several clinical parameters including age, duration of hospitalization, pulse rate, maximum blood pressure, erythrocyte sedimentation rate (ESR), blood urea nitrogen (BUN), neutrophil count, white blood cell (WBC) count, and oxygen saturation. However, we did not observe a significant association between CAC and the severity index of COVID-19. In addition, logistic regression tests did not find a significant value of CAC to predict in-hospital mortality.

**Conclusion:**

Our findings showed a significant relationship between CAC and in-hospital mortality.

In 2019, a new coronavirus known as severe acute respiratory syndrome coronavirus 2 (SARS-CoV-2) caused the COVID-19 pandemic (1). CoVID-19 causes various symptoms, ranging from asymptomatic to severe pneumonia, acute respiratory distress syndrome (ARDS), and death (2). The COVID-19 infection has been associated with myocardial damage, acute heart failure, shock, and arrhythmia (3-5). Thirty percent of patients with COVID-19 exhibit cardiac complications (6). Some studies suggest that the severity of the respiratory syndrome and the risk of complications increase in patients with a history of cardiovascular disease (7). 

Coronary heart disease (CHD) is one of the underlying cardiac conditions that has been suggested as a contributing factor to increasing COVID-19 mortality, which can be assessed by coronary artery calcification (CAC) (8, 9). In a study, the presence of CAC on the chest computed tomography (CT) scan of hospitalized COVID-19 patients was linked to a less favorable prognosis (10). CAC may be associated with increased ventilation requirements, and death independent to age, and atherosclerotic heart disease (11). 

However, further investigations are required to make a definite assumption. To create infection and enter to host cell, the SARS-CoV-2 virus needs to bind to the membrane receptor of angiotensin-converting enzyme 2 (ACE2) (12). In addition to other organs, the ACE2 receptor is also located in the heart (13). Consequently, cardiac complications of COVID-19 are prevalent, and potential associations between COVID-19 clinical outcomes and cardiovascular risk factors require discussion and investigation.

Although some studies have found relationships between cardiovascular diseases and COVID-19, the relationships between COVID-19 clinical outcomes and risk factors for cardiovascular diseases such as CAC and their measuring techniques are still under investigation. So, the goal of this study was to find out if there were potential links between CAC, clinical findings, COVID-19 severity, and the patient's in-hospital outcomes.

## Methods

This retrospective study evaluated 551 suspected patients admitted to teaching hospitals of Babol University of Medical Sciences, Babol, Iran, from March to October 2021. Among the patients with suspected clinical symptoms of COVID-19 who were referred to the emergency department and underwent a chest CT scan or PCR, 551 patients with a confirmed diagnosis of COVID-19 were hospitalized and underwent further evaluation.

In this study, all hospitalized COVID-19 patients were enrolled if they were over 18 and given informed consent to participate in the study. If the patients had any of the following conditions, they were excluded from the study: 1. history of diseases that interfere with calcium deposition, such as malignancies, chronic kidney disease (CKD) or end stage renal disease (ESRD); 2. history of cardiovascular disease; 3. presence of connective tissue diseases; such as Marfan’s syndrome and Ehlers-Danus; 4. any evidence for immunodeficiency; 5. and history of lung diseases.

This research was reviewed and approved by the Research Ethics Committee of Babol University of Medical Sciences (Ethics approval number 724133341), and written informed consent was obtained from the subjects to include the clinical details. Individuals' personal information remained confidential during data collection, transfer, and storage.

First, information was recorded about the symptoms of infectious disease and the possible history of receiving treatment for it. All the information related to the previous diseases, comorbidities, and history of the patient's medications were obtained from their medical records and accompanying documents and recorded in a checklist. Also, clinical examinations and routine laboratory tests including CBC Diff, BUN, Cr, Na, K, PT, PTT, INR, ESR, CRP, AST, ALT, ALP were performed for all patients. Demographic characteristics such as age, sex, duration of hospitalization, O2 saturation levels, need for ventilation, as well as the number of days under ventilation, in addition to laboratory data including the level of procalcitonin, ESR, CRP, D-dimer, troponin, interleukin-6, BUN, and creatinine were evaluated and recorded in a checklist. CAC was assessed using a chest CT scan (Siemens emotion 16 slice, Germany) reported by a radiologist.

SPSS Version 25 software was utilized to analyze data using t-test, ANOVA, chi-square, Spearman's correlation coefficient, and linear and logistic regression models. Results were reported using descriptive statistics, tables, and graphs. A p-value of less than 0.05 was considered significant.

## Results

52.3% of the patients were males, and 47.7% were females. In-hospital mortality was seen in 21 (3.8%) patients. 45 (8.2%) patients were admitted to ICU. CAC was significantly associated with in-hospital mortality (P = 0.003, OR = 1.311, CI: 0.710–2.420) but not with gender or ICU admission (P = 0.629 and P = 0.386, respectively) (table 1). 

**Table 1:** Relationship between CAC with gender, in-hospital mortality, and ICU admission

**Variable**		**Number (percentage)**	**Odds Ratio**	**95% CI**		**P-value**
				**Lower**	**Upper**	
**Gender**	Male	288 (52.3%)	1.086	0.777	1.517	0.629
	Female	263 (47.7%)				
**In hospital mortality**	Yes	21 (3.8%)	4.583	1.522	13.803	0.003
	No	530 (96.2%)				
**ICU admission**	Yes	45 (8.2%)	1.311	0.710	2.420	0.386
	No	506 (91.8%)				

*CAC: coronary artery calcification

CAC positive patients were 66.9 ± 15.6 years old and stayed in the hospital for 7.1 ± 5.6 days, while those without CAC were 52.1 ± 17.0 years old and stayed for 6.0 ± 4.8 days. Both groups significantly differed in age (p< 0.001) and duration of hospitalization (P = 0.024). The mean ICU admission days for CAC positive patients were 0.81 ± 3.1 days, while those without CAC were 0.71 ± 3.6 (P = 0.742) (table 2).

**Table 2:** Relationship between age, duration of hospitalization, and the number of days admitted to ICU with CAC

**Variable**	**CAC**		**P-value**
	**Yes (N = 272)**	**No (N = 279)**	
**Age**	66.9 ± 15.6	52.1 ± 17.0	< 0.001
**duration of hospitalization**	7.1 ± 5.6	6.0 ± 4.8	0.024
**Number of ICU admission days**	0.81 ± 3.1	0.71 ± 3.6	0.741

CAC: coronary artery calcification

The logistic regression test with gender and CAC control demonstrated that age perse increases in-hospital mortality (P = 0.031, OR: 1.037, CI: 1.003–1.072) (table 3). The logistic regression test with gender and CAC control also demonstrated that age perse increases in-hospital mortality (P = 0.031, OR: 1.037, CI: 1.003–1.072) (table 3).

Significant relationships between CAC and age (p< 0.001), duration of hospitalization (P = 0.024), pulse rate (P = 0.002), maximum blood pressure (P = 0.029), ESR (P = 0.007), BUN (P = 0.029), neutrophil count (P = 0.047), WBC count (P = 0.007), and O2 saturation levels (P = 0.012) were detected (table 4). In addition, CAC was not significantly associated with the COVID-19 severity index (P = 0.876). Table 5 and table 6 summarizes the significant and non-significant relationships between CAC and the studied variables in this investigation. 

**Table 3:** Logistic regression test to predict the in-hospital mortality of patients with age, gender, and CAC control

**Variable**	**Odds Ratio**	**95% CI**		**P-value**
		**Lower**	**Upper**	
**Age**	1.037	1.003	1.072	0.031
**Gender**	0.525	0.211	1.304	0.165
**CAC**	2.955	0.925	9.438	0.067

CAC: coronary artery calcification

**Table 4:** The relationship between CAC, and clinical and laboratory variables

**Variable**	**CAC**		**P-value**
	**Yes (N = 272)**	**No (N = 279)**	
**Age**	66.9 ± 15.6	52.1 ± 17.0	< 0.001
**duration of hospitalization**	7.1 ± 5.6	6.0 ± 4.8	0.024
**ICU admission days**	0.81 ± 3.1	0.71 ± 3.6	0.741
**Respiratory rate**	19.6 ± 5.2	19.6 ± 5.4	0.969
**Pulse rate**	86.6 ± 19.4	91.9 ± 18.9	0.002
**Max BP**	118.7 ± 21.6	115.0 ± 18.6	0.029
**Min BP**	76.0 ± 10.2	74.6 ± 8.7	0.148
**Troponin**	0.02 ± 0.127	0.02 ± 0.127	1.000
**ESR**	46.9 ± 31.4	39.3 ± 27.2	0.007
**BUN**	27.8 ± 17.7	19.8 ± 11.9	0.029
**PTT**	39.9 ± 19.5	41.4 ± 20.8	0.443
**PLT (× 1000)**	247.5 ± 102.2	256.0 ± 104.5	0.293
**Neut percent**	74.1 ± 14.8	74.2 ± 11.2	0.962
**Lymph percent**	19.6 ± 12.8	20.2 ± 9.9	0.562
**Neut count**	6505.2 ± 8247.5	5446.3 ± 3216.9	0.047
**Lumph count**	1980.1 ± 738.5	1435.2 ± 1386.2	0.230
**WBC count**	10158.3 ± 12601.8	7964.5 ± 3436.7	0.007
**O2 Saturation levels**	91.3 ± 6.6	92.6 ± 5.1	0.012
**Ventilation days**	3.0 ± 4.3	3.0 ± 4.1	0.994

CAC: coronary artery calcification

**Table 5:** A summary of significant relationships found between CAC and some variables

**Variables**	**P-value**
**Age**	< 0.001
**Duration of hospitalization**	0.024
**Pulse rate**	0.002
**Maximum blood pressure**	0.029
**BUN**	0.029
**WBC count**	0.007
**Neutrophil count**	0.047
**ESR**	0.007
**O2 saturation levels**	0.012

CAC: coronary artery calcification

**Table 6:** A summary of non-significant relationships found between CAC and some variables

Variables	P-value
Gender	0.629
ICU admission	0.386
Number of days admission in ICU	0.741
Respiratory rate	0.969
Minimum blood pressure	0.148
Troponin	1
PTT	0.443
Plt	0.293
Lymp Count	0.230
Percent of neut	0.962
Percent of lymph	0.562
Number of days under ventilation	0.994

PPT: Partial thromboplastin time, Plt: platelet, Lymp: Lymphocyte, Neut: Neutrophil

## Discussion

The goal of this study was to find out if there were potential links between CAC, clinical findings, COVID-19 severity, and in-hospital mortality. A significant relationship was found between CAC and in-hospital mortality, but our logistic regression test results did not find a significant value of CAC to predict in-hospital mortality.

Yogesh Sean Gupta et al. (14) conducted a similar study on 180 COVID-19 patients in 2021. Their results showed that CAC is significantly associated with intubation and in-hospital mortality. Although we did not find any significant relationship between a need for ventilation and CAC, in line with their conclusions, our results showed a significant link between CAC and in-hospital mortality.

Anirudh Venugopalan Nair et al. (15) conducted a study on 67 patients to assess the potential relationships between CAC and COVID-19 clinical outcomes in Qatar in 2021. Their results demonstrated a significant relationship between CAC and in-hospital mortality, assisted ventilation, and ICU admission. Similarly, we also found significant relationships between CAC and mortality but did not find statistically significant relationships between CAC and ventilation or ICU admission. However, we found a significant link between the duration of hospitalization and CAC. These inconsistencies may be due to differences in CAC measurement techniques and our hospitals' limited ICU beds.

In contrast to our results, Leandro Slipczuk et al. (16) conducted a study on 493 patients, and in their regression models, they found that CAC can be an independent predictor of mortality. We did not find any significant value in CAC for predicting mortality. However, there was a significant relationship between them. In 2021, Elie Mousseaux et al. (17) investigated the potential links between CAC and COVID-19 6-month mortality in 169 patients. Their results showed a significant relationship between these variables.

 Alberto Cereda et al. (18) investigated the possible links between gender, COVID-19 mortality, and CAC in 1683 patients. Their results showed that men had a significantly higher mortality rate and CAC than females. In contrast, we did not find significant relationships between gender and mortality. This could be due to the potential biases that occur when using a large sample size (19, 20).

In 2021, Philipp Fervers et al. (21) investigated the mortality prediction value of CAC (measured via the Agatston score) and other clinical variables in 89 patients. Their results demonstrated a significant relationship between mortality and CAC, creatinine, and leukocyte count. Similarly, we found the same relationships. However, we did not investigate the possible links between creatinine and mortality. Instead, we assessed the relationship between BUN and mortality, which was statistically significant. One of the limitations of the present study is the absence of a control group. Additionally, conducting further studies with a larger sample size is recommended to investigate the relationship.

 Our findings showed a significant relationship between CAC and in-hospital mortality. However, our logistic regression test results did not find a significant value of CAC to predict in-hospital mortality. CAC was also significantly related to age, duration of hospitalization, pulse rate, maximum blood pressure, ESR, BUN, neutrophil count, WBC count, and O2 saturation. However, it was not associated with the COVID-19 severity index.
